# Bound states in the continuum in divided triangular hole metasurfaces

**DOI:** 10.1038/s41598-024-63912-0

**Published:** 2024-06-06

**Authors:** Ruey-Lin Chern, Ti-Jung Hsu

**Affiliations:** Institute of Applied Mechanics, National Taiwan University, Taipei, 106 Taiwan

**Keywords:** Optics and photonics, Optical materials and structures, Metamaterials

## Abstract

We investigate the bound states in the continuum (BICs) in dielectric metasurfaces consisting of a two-part divided triangular hole in the unit cell of a square lattice, with emphasis on the generation, splitting, and merging of BICs. At the smallest height ratio between the upper triangular and the lower trapezoidal holes, the accidental BIC with an extremely large quality factor emerges on an isolated dispersion band at the Brillouin zone center, which is recognized as a polarization singularity (*V* point) with an integer topological charge. As the height ratio increases, the accidental BIC is split into a pair of circularly polarized states, which are polarization singularities (*C* points) with half-integer topological charges. The two states depart from each other to a maximum distance, and then approach each other as the height ratio continues to change. They finally merge to another polarization singularity (*V* point) with an integer topological charge, which is identified as the Friedrich-Wintgen BIC that occurs near the avoided crossing between two interacting dispersion bands.

## Introduction

Bound states in the continuum (BICs) are non-leaky localized resonance modes that coexist with a continuous frequency spectrum of radiating waves^1–4^. They are defined as the non-radiative eigensolutions of wave equation that are located above the light cone. As BICs will not radiate to the far field, they are decoupled from the far-field radiation. Ideal BICs possess infinite quality factors and zero spectral linewidths. In the presence of absorption loss, roughness, or finite size of the structure, ideal BICs may turn into quasi-BICs with finite, yet very large, quality factors and nonzero, yet very small, spectral linewidths. The prospect of BICs was first discovered in 1929 by von Neumann and Wigner in the context of quantum mechanics^5^. The essence of BICs was further elucidated in 1985 by Friedrich and Wintgen with the destructive interference between a pair of resonances through the evolution of continuous parameters in the system^6^. In addition to the quantum system, BICs have also been observed in photonic^7–11^, phononic^12^, and water wave^13^ systems. By virtue of the large quality factor nature, BICs can be applied in a variety of areas, including integrated photonic circuits^14^, filters^15^, lasers^10^, and biosensors^16^.

BICs occur in a number of photonic systems, such as gratings^17–19^, waveguides^20^, metasurfaces^21–23^, photonic crystals^24,25^, and photonic crystal slabs^26–31^. BICs are particularly useful in metasurfaces, which are ultrathin metamaterials composed of subwavelength microstructures exhibiting tailored electromagnetic responses. Interesting phenomena have been demonstrated in metasurfaces with broken in-plane inversion symmetry^32^. They are usually represented in the form of Fano resonance characterized by an asymmetric line shape due to interference between the scattering within a continuum state and an excitation of a discrete state. As a result, a transmission (or reflection) peak is accompanied with a transmission (or reflection) dip at a nearby frequency. Fano resonance is considered a precursor of BICs^33^, which becomes sharper and sharper and eventually disappears when its quality factor goes to infinity, and the resonance mode turns into a BIC^34^. A special Fano resonance occurs when the frequencies of strongly and weakly damped oscillators match, resulting in a narrow transparency window known as the electromagnetic-induced transparency (EIT)^33^. As metamaterials usually suffer from high loss due to strong resonances, metasurfaces with large quality factors offer a new platform to control light waves^35^. BICs in metasurfaces are especially suitable for applications in ultrasensitive sensors, enhanced light emission, perfect absorbers, and nonlinear optics with strong localization of electromagnetic energy^36^.

There are in general two categories of BICs. The first one is symmetry-protected (SP) BICs due to symmetry mismatch between resonance modes and incident waves, which usually appear at the center of Brillouin zone for a periodic lattice with perfect symmetry in structure geometry^37,38^. The SP BICs have been identified in the metasurfaces composed of arrays of meta-atoms such as tilted elliptic strip pairs^39,40^, nanodisks with holes^41^, circular split rings^42^, rectangular blocks^43^ or strip pairs^44–46^, square split rings^47^, and asymmetric dual patches^23^. The second one is accidental BICs originating from wave interference between two or more radiation channels, which usually appear at certain wave vector points on *isolated* dispersion bands (at or off the Brillouin zone center) when the relevant coupling to the radiation continuum completely vanishes^26^. Single-resonance parametric BICs^1^ and resonance-trapped BICs^48^ are also regarded as the accidental BICs. This category of BICs can further be divided into two types: Fabry-Perot (FP) BICs^49,50^ and Friedrich-Wintgen (FW) BICs^51^. The former are constructed by two interacting objects with one resonance, while the latter by two interacting resonances in one object^1^. In particular, FW BICs are generally found in the vicinity of *avoided crossing* between two dispersion bands, which arise because of the destructive interference of two resonances coupled to the same radiation channel^52^. In practice, accidental BICs can be created by deviating the cross-shaped structure^53^, and FW BICs by tuning the geometric parameters of split-ring structures^54–56^.

Radiation polarization states of the eigenmodes on a dispersion band defines a polarization field in the momentum (wave vector) space. The far-field polarization vectors of BICs possess intriguing topological natures^28^, which are revealed by the spatial patterns of polarization singularities, including *V* points (vortex centers of polarization fields), *C* points (points of circular polarization), and *L* lines (curves of linear polarization)^57^. Various types of BICs such as SP BICs, accidental BICs, and FW BICs, appear as vortex centers in the polarization states of far-field radiation. A remarkable feature of the vortex centers is that they carry integer topological charges^58^. As the topological charge is a conserved quantity, the existence of BICs is robust against small disturbances of the system parameters, provided that the disturbances do not change the topology^28^. In addition to vortex centers with integer topological charges ($q=\pm 1, \pm 2, \dots$), circularly polarized states are polarization singularities with half-integer topological charges ($q=\pm 1/2$). The latter are resonances with finite, usually not significant, quality factors that are coupled to circularly polarized radiative plane waves^59^. Note that the integer topological charge associated with the BIC could be zero in a certain situation when the annihilation of half-integer topological charges with opposite sign occurs^60^.

By breaking the structure symmetry, the splitting of a BIC may occur. For instance, a SP BIC can split into a pair of *C* points with right-handed and left-handed circular polarizations^59,61,62^. A similar splitting phenomenon also occurs for an accidental BIC^60^. A SP BIC can even split into a group of accidental BICs^28^. A SP BIC as a high-order *V* point ($q=\pm 2$) can split into a pair of accidental BICs as low-order *V* points ($q=\pm 1$)^59,63^. In addition to the splitting of a SP or an accidental BIC, a pair of *C* points may merge to form an accidental BIC^60,62^. A SP BIC and a number of accidental BICs can merge to form another SP BIC^28,29^. A SP BIC as a high-order *V* point and a number of accidental BICs may merge to form a SP BIC as another high-order *V* point^63^. A FW BIC and an accidental BIC can merge to form a new accidental BIC^30^. Occasionally, the splitting and merging of BICs may occur in the same structure^60,62^.

**Figure 1:**
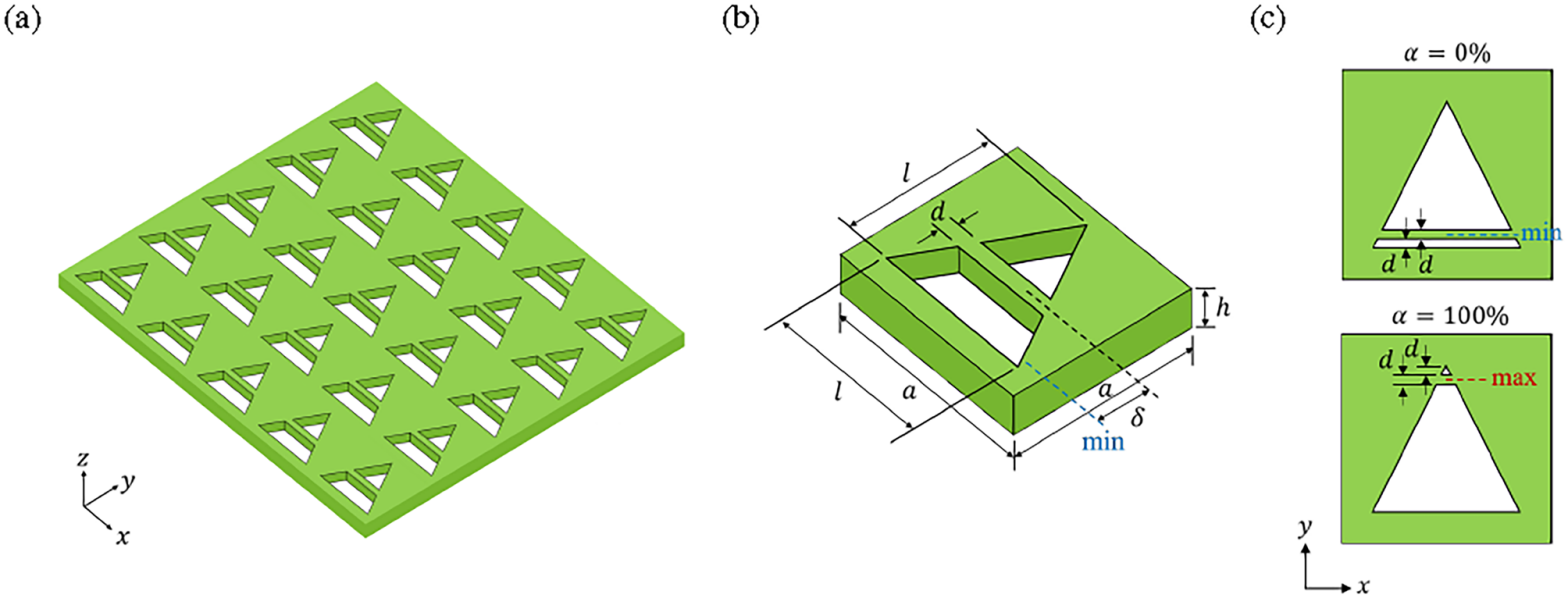
Schematic diagram of the two-part divided triangular hole metasurface. (**a**) Square lattice, (**b**) unit cell, (**c**) smallest height ratio (upper plot) and largest height ratio (lower plot) between upper triangular hole and lower trapezoidal hole. Blue and red dashed lines indicate the bridge center positions for minimum and maximum $\delta$, respectively.

In the present study, we investigate the BICs in two-part divided triangular hole metasurfaces, with emphasis on the generation, splitting, and merging of BICs. It has been shown in a previous study that circularly polarized states are generated from a SP BIC by breaking $C_2$ symmetry in dielectric metasurfaces through deforming square holes to isosceles triangular holes^61^. Here, we introduce a horizontal dielectric bridge in the triangular hole (the most asymmetric case in the above structure), such that the unit cell is divided into two parts: an upper triangular hole and a lower trapezoidal hole. For this structure, a height ratio parameter between the two holes is defined for measuring an extra asymmetry in the two-part triangular hole structure. By tuning the height ratio parameter, the splitting from and the merging to a BIC may occur in the same structure. In particular, an accidental BIC with an extremely large quality factor emerges on an isolated dispersion band at the smallest height ratio, which appears as a vortex center (*V* point with $q=-1$) of the polarization states at the Brillouin zone center ($\Gamma$ point). The accidental BIC is split into a pair of circularly polarized states (*C* points with $q=-1/2$) with opposite handedness as the height ratio increases from a small value. The pair of *C* points depart from each other to a maximum distance at a medium height ratio. As the height ratio continues to change, the pair of *C* points approach each other and merge to form another vortex center (*V* point with $q=-1$) at the $\Gamma$ point, which is identified as the FW BIC with an even larger quality factor, featured with the avoided crossing between two interacting dispersion bands.

## Results

### Theory and model design

Consider a dielectric metasurface of thickness *h* consisting of a two-part divided triangular hole in the unit cell of a square lattice of period *a*, as schematically shown in Fig. 1. An isosceles triangular hole of base *l* and height *l* is divided by a horizontal dielectric bridge of width *d* into two parts: a upper isosceles triangular hole and a lower trapezoidal hole (cf. Fig. 1b). In this configuration, a height ratio parameter $\alpha$ is defined as $\alpha \equiv \delta /\delta _{\textrm{max}}$ for measuring the relative sizes of the upper and lower holes, where $\delta$ is the distance from the bridge center to its lowest position and $\delta _{\textrm{max}}$ is the maximum value of $\delta$. Here, $\alpha =0\%$ is the smallest value when the height of lower trapezoidal hole is equal to the bridge width, while $\alpha =100\%$ ($\delta =\delta _{\textrm{max}}$) is the largest value when the height of upper triangular hole is equal to the bridge width (cf. Fig. 1c). The height ratio parameter is similar to the asymmetry parameter introduced in the metasurfaces composed of asymmetric split-ring resonators^54^ or asymmetric dual patches^23^, which serves as a system parameter to be tuned for the formation of BICs. In the present study, *a* = 450 nm, *h* = 100 nm, *l* = 315 nm, and *d* = 20 nm are used as the basic geometric parameters for the metasurface, and $\delta$ = 0 nm ($\alpha =0\%$) corresponds to the case of lowest bridge position and $\delta$ = 255 nm ($\alpha =100\%$) to the case of highest bridge position.

**Figure 2:**
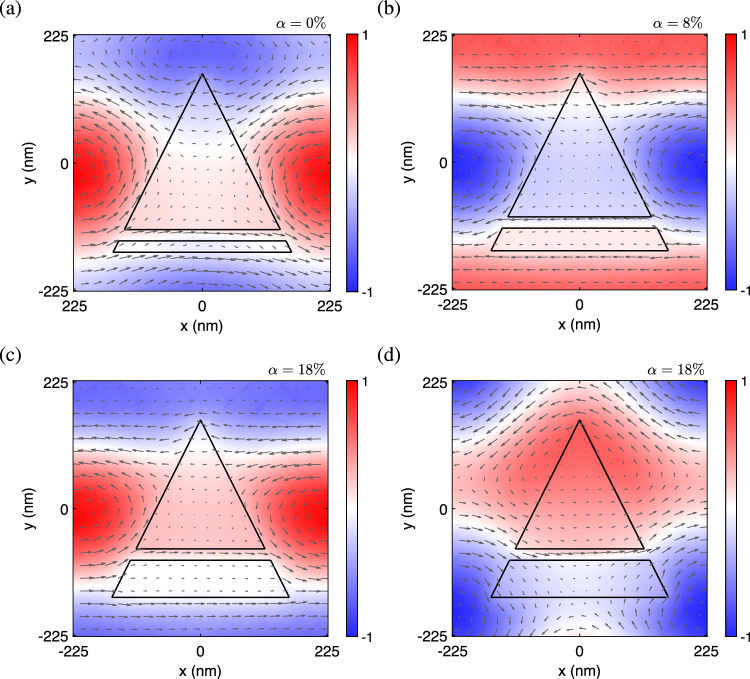
Resonance modes for the formation of BICs in the two-part divided triangular hole metasurface. Out-of-plane magnetic fields (Re[$H_z$]) in color and in-plane displacement currents (Re[$\partial \textbf{D}/\partial t$]) by arrows for (**a**) accidental BIC at $\alpha = 0\%$, (**b**) *C* point at $\alpha = 8\%$, (**c**) Friedrich-Wintgen BIC at $\alpha = 18\%$, (**d**) resonance counterpart for the FW BIC in (**c**).

### Resonance modes for the formation of BICs

Resonance modes are usually leaky when they are located in the continuous frequency spectrum^1^. BICs are a special type of resonance modes that are non-leaky, which may occur when the structure is judiciously designed with suitable parameters to be tuned. Figure 2 shows the resonance modes illustrated with the patterns of out-of-plane magnetic fields (Re[$H_z$]) and in-plane displacement currents (Re[$\partial \textbf{D}/\partial t$]), which are responsible for the formation of BICs in the present study. In particular, the field pattern in Fig. 2a corresponds to the accidental BIC (*V* point) at $\alpha =0\%$. The field pattern in Fig. 2b is associated with one of two circularly polarized states (*C* points) at $\alpha =8\%$. The field pattern of the other circularly polarized state is very much alike, except that the handedness is opposite. The field pattern in Fig. 2c corresponds to the FW BIC at $\alpha =18\%$. For completeness, the field pattern of the resonance counterpart for the FW BIC is given in Fig. 2d, both of which occur near the avoided crossing between two interacting dispersion bands. The above features will be revisited in the later section. Note that the resonance modes for the formation of BICs in the present structure are quadrupole-like modes with even symmetry in either the vertical or horizontal direction. In other design of metasurfaces that support the formation of BICs, the corresponding resonance modes can be the more common dipole-like modes with odd symmetry, or the LC-like modes, also known as the magnetic dipole-like modes, with circulating displacement currents over the unit cell^23,54,55^.

**Figure 3:**
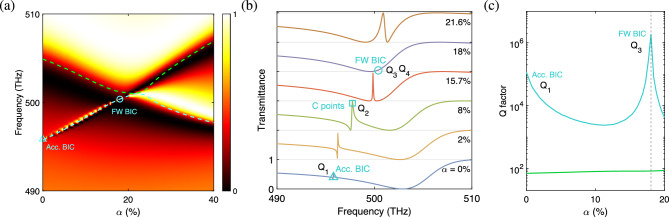
Accidental and Friedrich-Wintgen BICs in the two-part divided triangular hole metasurface. (**a**) Transmittance diagram as a function of the height ratio parameter $\alpha$ and the frequency. (**b**) Transmittance curves as functions of the frequency for selected $\alpha$. (**c**) Quality factors of two relevant dispersion bands as functions of $\alpha$.

### Accidental BIC

Figure 3a shows the transmittance diagram of the two-part divided triangular hole metasurface as a function of the height ratio parameter $\alpha$ and the frequency (cf. **Results: Theory and model design.**). The dispersion bands are overlaid in the same diagram to show consistent trends with the transmittance. There are two relevant dispersion bands (green and blue curves) that are associated with the BICs. In Fig. 3b, the transmittance curves as functions of the frequency (with interval $\Delta f=0.05$ Hz) are plot for selected height ratios. At $\alpha = 0\%$, where the dielectric bridge is at its lowest position (cf. Fig. 1c), a BIC occurs on the lower dispersion band, which is identified as the accidental BIC. In particular, the linewidth vanishes ’accidentally’ at the frequency $f=495.75$ THz, where the transmittance curve tends to be flat with a tiny ripple (cf. point $Q_1$ on the lowest curve in Fig. 3b), and a very large quality factor (on the order of $10^5$) is attained at its peak, as shown in Fig. 3c. This is characteristic of the accidental BIC^53^ that emerges on an isolated band because of the destructive interference between two or more leakage channels. In the present configuration, the accidental BIC is a quadrupole-like mode (cf. Fig. 2a).

### Friedrich-Wintgen BIC

As $\alpha$ increases, the tiny ripple on the transmittance curve at $\alpha =0\%$ gradually grows to a Fano resonance, a type of resonant scattering phenomenon that gives rise to an asymmetric line shape. For this resonance, a transmission peak is accompanied nearby with a transmission dip. The Fano resonance becomes apparent at $\alpha =2\%$ and a significant Fano resonance appears at $\alpha = 8\%$, where the accidental BIC is split into two circularly polarized states (cf. point $Q_2$ on the fourth curve in Fig. 3b). This feature will be made clear in the later section. At $\alpha =15.7\%$, the Fano resonance becomes an electromagnetic induced transparency (EIT), which is a special case of Fano resonance when the frequencies of strongly and weakly damped oscillators match and the Fano parameter vanishes^33^. A symmetric line shape is shown in the corresponding transmittance curve (cf. third curve in Fig. 3b). As $\alpha$ continues to increase, the frequency of the upper (lower) band is reduced (raised) and the two bands approach each other to form an avoided crossing at $\alpha =22\%$ ($f=500.99$ THz). This feature will be illustrated with the band structure in the later section. Near the avoided crossing point, the linewidth of the lower mode (blue curve) vanishes at $\alpha =18\%$ (cf. point $Q_3$ on the second curve in Fig. 3b) and an even larger quality factor (on the order of $10^6$) is attained at its peak ($f=500.38$ THz) (cf. Fig. 3c). The even larger quality factor of the resonance mode is related to the merging of two circularly polarized state. The merging feature will be revisited in the later section. Meanwhile, the quality factor of the upper mode (green curve) is much smaller (cf. Fig. 3c). The above feature is characteristic of the FW BICs^53,54^ formed by tuning the interaction between two resonances. In the present study, the two interacting resonances for the FW BIC are also quadrupole-like modes (cf. Fig. 2c, d). As $\alpha$ continues to change, the two dispersion bands depart from each other without further interactions.

The property of FW BICs can be understood from the coupled mode theory for a simple system with two resonances: $i{\partial A}/{\partial t}={{\mathcal {H}}}A$^1^, where $A=\left( A_1,A_2\right) ^T$ are the amplitudes of resonances and

1 $$\begin{aligned} H = \left( {\begin{array}{*{20}{c}} {{\omega _1}} &{} \kappa \\ \kappa &{} {{\omega _2}} \\ \end{array}} \right) - i\left( \begin{array}{*{20}{c}} {{\gamma _1}} &{} {\sqrt{{\gamma _1}{\gamma _2}} } \\ {\sqrt{{\gamma _1}{\gamma _2}} } &{} {{\gamma _2}} \\ \end{array} \right) \end{aligned}$$

is the Hamiltonian of the system. Here, $\omega _i$ and $\gamma _i$ ($i = 1, 2$) are the resonant frequencies and damping rates, respectively, of the *i*-th resonance, and $\kappa$ is the coupling strength between the two resonances. If the following condition is satisfied^1^:

2 $$\begin{aligned} \kappa ({\gamma _1} - {\gamma _2}) = \sqrt{{\gamma _1}{\gamma _2}} ({\omega _1} - {\omega _2}), \end{aligned}$$

the eigensystem for the Hamiltonian *H* is solved to give a pair of eigenvalues: ${\omega _ r } = \left( {{ {\gamma _1}{\omega _2}- {\gamma _2}{\omega _1} }}\right) /\left( {{{\gamma _1} - {\gamma _2}}}\right)$ and ${\omega _c } = \left( {{{\gamma _1}{\omega _1} - {\gamma _2}{\omega _2}}}\right) /\left( {{{\gamma _1} - {\gamma _2}}}\right) - i\left( {{\gamma _1} + {\gamma _2}} \right)$. The former is a purely real number and the corresponding eigenmode turns into a BIC without any loss, while the latter is a complex number and the corresponding eigenmode becomes more lossy, with the imaginary part equal to the sum of two damping rates. The above feature is characteristic of the FW BICs^6^, which is valid when ${\omega _1} \approx {\omega _2}$ and ${\gamma _1} \approx {\gamma _2}$ [cf. Eq. (2)], that is, both the frequencies and damping rates of the two resonances are roughly equal. This condition also gives a clue for tuning the system parameter to seek for the existence of FW BICs when a real structure is taken into account. In the present problem, the FW BIC occurs at the height ratio $\alpha =18\%$ (cf. Fig. 3), where the areas of upper triangular hole and lower trapezoidal hole are sufficiently close with a relative difference of $5\%$. However, the fact that two holes have comparable areas provides a clue rather than a rigorous explanation for the existence of a FW BIC.

**Figure 4:**
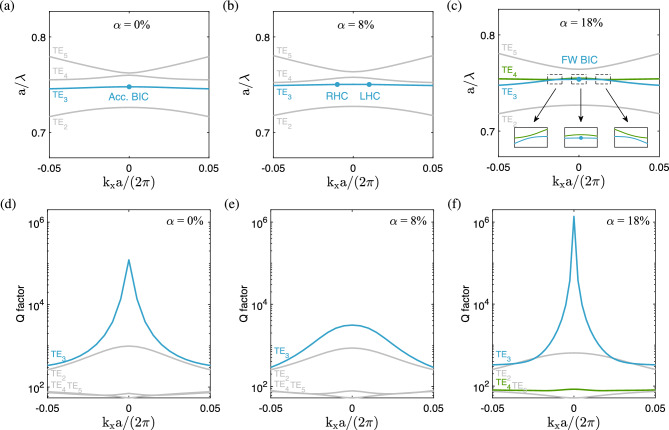
Projected band structures (at $k_y=0$) and corresponding quality factors for the two-part divided triangular hole metasurface. (**a, d**) Accidental BIC at $\alpha = 0\%$ (**b**) and (**e**) circularly polarized states at $\alpha = 8\%$ (**c, f**) Friedrich-Wintgen BIC at $\alpha = 18\%$.

### Dispersion bands and quality factors

Figure 4 shows four relevant dispersion bands and quality factors of the two-part divided triangular hole metasurface along the $k_x$ axis in the first Brillouin zone for three height ratios. In Fig. 4a, where $\alpha =0\%$, the accidental BIC occurs on the third band of transverse electric (TE) modes at $k_x=0$. Here, TE refers to the case where the electric fields are perpendicular to the *z* axis, that is, $E_z=0$. In the present study, the transverse magnetic (TM) modes, where the magnetic fields are perpendicular to the *z* axis, appear at a higher frequency range, which is outside the scope of our main focus. Recall that the field patterns of resonance modes for the formation of BICs are illustrated with out-of-plane magnetic fields and in-plane displacement currents (Fig. 2), a typical representation of TE modes. The accidental BIC appears ’accidentally’ on an isolated band, with the quality factor growing to an extremely large value (on the order of $10^5$) at $k_x=0$, as shown in Fig. 4d. Specifically, the quality factor is shown to decay quadratically ($Q\propto 1/k^2$) with respect to the distance *k* from a single isolated BIC^29^. Meanwhile, the other bands have much smaller quality factors.

In Fig. 4b, where $\alpha =8\%$, there are two circularly polarized states (*C* points) on the third band, symmetrically located at a distance from $k_x=0$, which is considered a result of splitting from the accidental BIC at $\alpha =0\%$. In the present configuration, the splitting of an accidental BIC is attained by adding an extra asymmetry in the system with broken $C_2$ symmetry through the height ratio parameter. Introducing a horizontal bridge in the hole does not actually induce further asymmetry in the system, but provides a new parameter for an additional degree of freedom to change the behaviours of polarization singularities. The right-handed state (with $s_3=1$) and left-handed state (with $s_3=-1$) are positioned on the negative and positive $k_x$ axes, respectively. The two *C* points are not BICs, for the corresponding quality factors are much smaller, as shown in Fig. 4e. The splitting of an accidental BIC into two circular polarized states has been observed in misaligned gratings^60^. A similar splitting feature of a BIC was reported in dielectric metasurfaces^59,61^ and magnetic gratings^62^, except that the corresponding BIC is a SP BIC instead of an accidental BIC. In Fig. 4c, where $\alpha =18\%$, the third band form an avoided crossing^6,53,70^ with the fourth band near $k_x=0$ (cf. enlarged view of frequency bands in the inset), at which the FW BIC occurs (cf. blue dot in the inset). The two bands approach each other but will not cross as the resolution of $k_x$ is increased. The avoided crossing feature is most significant at $k_y=0$. As $|k_y|$ increases, the avoided crossing becomes less evident. The quality factor for the FW BIC grows to an even larger value (on the order of $10^6$), one order magnitude larger than that of the accidental BIC, as a consequence of the merging from two circularly polarized states, whereas the quality factor of the resonance counterpart for the FW BIC (on the fourth band) is much smaller, as shown in Fig. 4f. In the present configuration, the merging of two *C* points is attained by continuously adding an extra asymmetry in the system with broken $C_2$ symmetry through the height ratio parameter. The scaling of quality factor changes to a higher decay order as in the merging of BICs^29,63^. A similar feature of the merging from two circularly polarized states to form a BIC has been reported in grating structures^60,62^, except that the corresponding BIC is an accidental BIC instead of a FW BIC.

**Figure 5:**
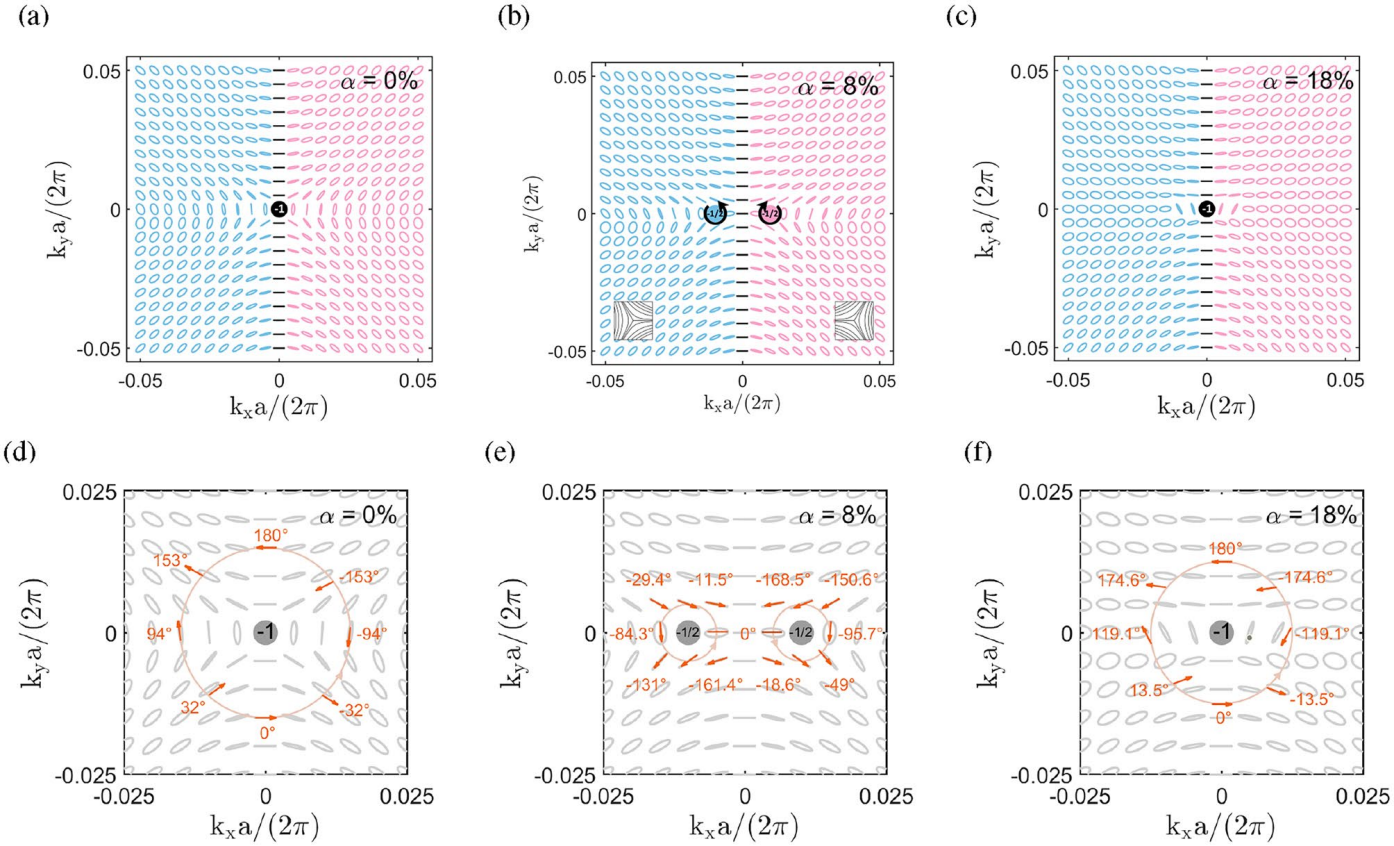
Polarization states and topological charges for the two-part divided triangular hole metasurface. (**a, d**) Accidental BIC at $\alpha = 0\%$, (**b, e**) circularly polarized states at $\alpha = 8\%$, (**c, f**) Friedrich-Wintgen BIC at $\alpha = 18\%$. Blue and red colors in (**a–c**) correspond to right-handed and left-handed elliptical or circular polarizations, respectively.

### Polarization states and topological charges

Figure 5 shows the polarization maps and topological charges of the third dispersion band for the two-part divided triangular hole metasurface at three height ratio parameters. The polarization states are represented by ellipses determined by the rotation angle $\psi$ and the ellipticity angle $\chi$ based on the Stokes parameters (cf. **Results: Polarization states.**). Figure 5 shows the polarization map constructed by the polarization ellipse at each wave vector point in the first Brillouin zone. The polarization states have reflection symmetry about the vertical direction and roughly reflection symmetry about the horizontal direction. In Fig. 5a, where $\alpha =0\%$, the orientations of polarization ellipses largely conform to the distribution $\phi (\textbf{k})\propto k_y+ik_x$, which is the $B_2$ representation in the symmetry group $C_{4v}$^63^. Except for a very small region along the vertical line at $k_x=0$, where the polarizations are linear, the electromagnetic waves are in general elliptically polarized with nonzero ellipticity angles, which is considered a consequence of broken $C_2$ symmetry^61^.

In this configuration, a singularity is located at the center of Brillouin zone, where the polarization state cannot be defined. The polarization singularity appears as a vortex center (*V* point) that corresponds to the accidental BIC (cf. Fig. 4a). The topological nature of a BIC is manifest on the existence of a nonzero topological change. In Fig. 5d, the polarization vectors circulate around the *V* point by 360^∘^ in the clockwise direction (see supplementary video: accidental BIC), giving rise to a topological charge $q=-1$ (cf. **Results: Topological charges.**). This feature is consistent with the fact that the topological charge *q* is an integer if the loop *C* [cf. Eq. (3)] encloses a *V* point, where ${s_1}$, ${s_2}$, ${{s_3} = 0}$^1,28^. Generally, *V* points represent BICs in the system with ${\sigma _z}$ symmetry, where no radiation channels are allowed, although the mode exists within the light cone^60^.

In Fig. 5b, where $\alpha =8\%$, the polarization map is similar to that at $\alpha =0\%$, except that there are two polarization singularities symmetrically located on the $k_x$ axis at a distance from the Brillouin zone center. The two polarization singularities are circularly polarized states (*C* points) with the ellipticity angle $\psi =\pm 45^\circ$, which are recognized as the ’star’ type singularities^58^, as schematically shown in the insets. The two *C* points are considered split from the accidental BIC (*V* point) at $\alpha =0\%$ by increasing the height ratio parameter. It is shown in Fig. 5e that the polarization vectors circulate around each *C* point by 180^∘^ in the clockwise direction (see supplementary video: circularly polarized states), giving rise to a topological charge $q=-1/2$. This feature is consistent with the fact that the topological charge *q* is a half integer if the loop *C* [cf. Eq. (3)] encloses a circularly polarized state, where ${s_1} = {{s_2} = 0}$ and ${s_3} = \pm 1$^61,71^. As the topological charge is a conserved quantity, the sum of two charges for the circularly polarized states is equal to that of the accidental BIC, that is, $\left( -1/2\right) +\left( -1/2\right) =-1$. In Fig. 5c, where $\alpha =18\%$, a polarization singularity appears again at the center of Brillouin zone as a vortex center or *V* point, with a similar polarization vector distribution as in Fig. 5a. This *V* point is a FW BIC characterized by the avoided crossing between the third and fourth bands (cf. Fig. 4c), and is considered a result of merging from two circularly polarized states. The polarization vectors circulate around the *V* point by 360^∘^ in the clockwise direction (see supplementary video: Friedrich-Wintgen BIC), giving rise to a topological charge $q=-1$, as shown in Fig. 5f. The topological charge of the FW BIC is equal to the sum of two charges for the circularly polarized states, that is, $-1=\left( -1/2\right) +\left( -1/2\right)$, as a result of the conservation of topological charge. The splitting from an accidental BIC to a pair of *C* points, followed by the merging of two *C* points to form a FW BIC is achieved by increasing the height ratio parameter in the structure (see supplementary video: splitting and merging of BIC). The above feature does not occur for the second band, where the polarization pattern is completely different, that is, a singularity does not exist at the $\Gamma$ point and the corresponding topological charge is zero.

## Methods

### Transmittance

To analyze the basic properties of BICs, we calculate the transmittance (the ratio of transmitted power to incident power) based on the finite element method, which has been employed to study the transmission characteristics of surface structures with patches or apertures^23,64^. In the present problem, the dielectric material is chosen as $\text{{S}}{\text{{i}}_3}{\text{{N}}_4}$ with the refractive index 2.02^61^. The incident wave is normal to the metasurface and polarized parallel to the horizontal bridge, for the horizontal polarization delivers more significant features of BICs than the vertical polarization in the present configuration^23^.

### Eigenstates

To identify various types of BICs, we compute the eigenfrequencies based on the plane wave expansion and the supercell method^65,66^. An important factor for evaluating BICs is the quality factor (*Q* factor) based on the eigenfrequency $\Lambda$, which is defined as $Q=\textrm{Re}[\Lambda ]/\left( 2\textrm{Im}[\Lambda ]\right)$^67^, where $\textrm{Re}[\cdot ]$ and $\textrm{Im}[\cdot ]$ denote the real and imaginary parts, respectively. Another definition of the quality factor is based on the spectral response as $Q=f_r/\Delta f_r$, where $f_r$ is the resonant frequency and $\Delta f_r$ is the linewidth (full width at half maximum) of the transmittance (or reflectance). The above two definitions of the quality factor are nearly equivalent and amount to the ratio of energy stored in the oscillating resonator to the energy dissipated per cycle by damping processes^68^. A larger quality factor corresponds to a narrower linewidth with a smaller damping rate and a longer lifetime for the resonance. Theoretically, the linewidth of an ideal BIC becomes zero and the quality factor tends to infinity^1,32^.

### Polarization states

The BICs are usually located at the polarization singularities, at which the polarization states in the far field cannot be defined^60,61^. The polarization state of an electromagnetic wave can be described by the Stokes parameters $S_i$, which are given by $S_0=E_xE_x^*+E_yE_y^*$, $S_1=E_xE_x^*-E_yE_y^*$, $S_2=E_xE_y^*-E_yE_x^*$, and $S_3=i\left( E_xE_y^*-E_yE_x^*\right)$. The polarization ellipse traced out by the electric field for an electromagnetic wave of general elliptical polarization is determined by the rotation angle $\psi =\frac{1}{2}\tan ^{-1}\left( S_2/S_1\right)$ and the ellipticity angle $\chi =\frac{1}{2}\tan ^{-1}\left( S_3/\sqrt{S_1^2+S_2^2}\right)$^69^. The former is the angle between the major axis of the ellipse and a reference direction (the *x* axis), a measure of the ellipse orientation. The latter is the angle determined by the length ratio of the semi-minor axis to the semi-major axis, a measure of the ellipse eccentricity. A polarization map can thus be generated by plotting the polarization ellipse at each wave vector point in the momentum space.

### Topological charges

The polarization singularities usually carry nonzero topological charges^60^, which are defined as the number of times the polarization vectors wind around the singularity^28,59,60^:

3 $$\begin{aligned} q {{ = }}\frac{{{1}}}{{{{2}}\pi }}\oint _C {d{k_\parallel }\cdot {\nabla _{{k_\parallel }}}\phi \left( {{k_\parallel }} \right) }, \end{aligned}$$

where ${{{k}_\parallel }=k_x {{\hat{x}}}+k_y {{\hat{y}}}}$, $\phi \left( {{k_\parallel }} \right) = \frac{1}{2}\arg \left[ {{S_1}({k_\parallel }) + i{S_2}({k_\parallel })} \right]$ is the angle between the long axis of polarization ellipse and the *x* axis (equivalent to the rotation angle $\chi$), ${S_i}({k_\parallel })$ is the Stokes parameter of $\textbf{d}\left( {{k_\parallel }} \right) = {d_x}\left( {{k_\parallel }} \right) {\hat{x}} + {d_y}\left( {{k_\parallel }} \right) {\hat{y}}$, the projection of polarization vector of the far-field radiation onto the *xy* plane, and *C* is a closed path that goes around the polarization singularity in the counterclockwise direction.

In conclusion, we have investigated the BICs in dielectric metasurfaces consisting of a two-part divided triangular hole in the unit cell of a square lattice. An accidental BIC with an extremely large quality factor appears on an isolated dispersion band at the smallest height ratio between the upper triangular and the lower trapezoidal holes, which corresponds to a polarization singularity (*V* point) with an integer topological charge. The accidental BIC is split into two circularly polarized states (*C* points), each with a half-integer topological charge, as the height ratio increases. The two *C* points are then merge to form a FW BIC near the avoided crossing between two interacting bands, with an even larger quality factor, as the height ratio parameter is further increased. The full process in the generation of, splitting from, and merging to a BIC is fulfilled in the present structure by tuning a single system parameter. A brief summary in this aspect with a comparison to two previous literatures as a final remark is given in Table 1.

**Table 1:** Summary of the generation, splitting, and merging of BICs.

Structure	Generation	Splitting	Merging
Grating^62^	Symmetry-Protected BIC	Circularly polarized states	Accidental BIC
Grating^60^	Accidental BIC	Circularly polarized states	Accidental BIC
Metasurface (present work)	Accidental BIC	Circularly polarized states	Friedrich-Wintgen BIC

### Supplementary Information

Supplementary Information 1.

Supplementary Information 2.

Supplementary Information 3.

Supplementary Information 4.

## Data Availability

All data generated or analysed during this study are included in this published article.
